# China’s human resources for maternal and child health: a national sampling survey

**DOI:** 10.1186/s12913-015-1238-9

**Published:** 2015-12-16

**Authors:** Zhenghong Ren, Peige Song, Evropi Theodoratou, Sufang Guo, Lin An

**Affiliations:** Department of Child, Adolescent and Women’s Health, School of Public Health, Peking University, Beijing, China; Centre for Population Health Sciences, the University of Edinburgh, Edinburgh, UK; UNICEF China, Beijing, China

**Keywords:** Maternal and child health, Human resources, China

## Abstract

**Background:**

In order to achieve the Millennium Development Goals (MDG) 4 and 5, the Chinese Government has invested greatly in improving maternal and child health (MCH) with impressive results. However, one of the most important barriers for further improvement is the uneven distribution of MCH human resources. There is little information about the distribution, quantity and capacity of the Chinese MCH human resources and we sought to investigate this.

**Methods:**

Cities at prefectural level were selected by random cluster sampling. All medical and health institutions providing MCH-related services in the sampled areas were investigated using a structured questionnaire. The data were weighted based on the proportion of the sampled districts/cities. Amount, proportions and numbers per 10,000 population of MCH human resources were estimated in order to reveal the quantity of the Chinese MCH human resources. The capacity of MCH human resources was evaluated by analyzing data on the education level and professional skills of the staff.

**Results:**

There were 77,248 MCH workers in China in 2010. In general, 67.6 % and 71.9 % of the women’s and children’s health care professionals had an associate degree or higher, whereas around 30 % had only high-school or lower degrees. More than 40 % of the women’s health workers were capable of providing skilled birth attendance, but these proportions varied between different institutions and locations.

**Conclusions:**

Evidence from this study highlights that Chinese MCH human resources are not in shortage in the national level. However, the quantity and capacity of MCH human resources are not evenly distributed among different institutions and locations. Finally there is a need in the improvement of the MCH services by improving the quality of MCH human resources.

## Background

Health of women and children is the main foundation of sustained human development and a high priority for the international community [1, 2]. In 2000, 189 member states of United Nations adopted the Millennium Declaration committing to achieve 8 Millennium Development Goals (MDGs), aiming to accelerate development and to reduce poverty. Of these goals, two are directly related to maternal and child health: MDG 4 (a two-thirds reduction in under-5 mortality between 1990 and 2015) and MDG 5 (a three-quarters reduction in maternal mortality ratio between 1990 and 2015) [3]. Since then, a number of efforts have been taken to globally reduce child mortality and to improve maternal health. One such effort is the “Countdown to 2015” project, a global initiative that began in 2003 to track progress in maternal, newborn and child health in the 75 highest burden countries and to promote action and accountability [4].

In addition to these international efforts, there are several domestic initiatives. The Chinese Government has invested greatly in improving maternal and child health (MCH) by promoting institutional delivery and implementing public MCH service programs [5]. One of these programs is a national program called “Reduce Maternal Mortality and Eliminate Neonatal Tetanus”, which was implemented in 2000 and provided obstetric equipment for hospitals and training of medical staff. In the end of 2011, this program reached a coverage of 2,297 counties (80.5 % of total) and of around 830 million people (60.6 % of total) [1]. Additional national efforts include the implementation of a national healthcare system for women by increasing governmental financial input and by incorporating maternity insurance into the social security system [6]. In the past decade, the proportion of mothers who have had five or more antenatal visits during pregnancy increased from 43.2 % in 2003 to 62.8 % in 2011, and institutional delivery rates rose from 73.3 % in 2003 to 95.8 % in 2011, all of which indicate a big acceleration in promoting MCH in an effort to reduce maternal and child mortality [7].

China has been considered as one of the countries that are set to achieve both MDGs 4 and 5 by 2015 [8, 9]. In particular, the under-5 mortality reduced from 45.7 and 13.8 (per 1000 live births) in rural and urban areas in 2000 to 19.1 and 7.1 in 2011, and the maternal mortality reduced from 69.6 and 29.3 (per 100,000 live births) in rural and urban areas in 2000 to 26.5 and 25.2 in 2011. However, inequalities in MCH between urban and rural areas, between different regions and between different population groups still exist and the overall development of MCH service network is lagging behind [1].

Health human resources refer to all professionals engaged in health works, which normally include physicians, nurses and midwives, as well as dentists and pharmacists, laboratory technicians, etc. [10, 11]. Estimation of the human resources can help to identify the constraints and the potential impacts on population health. Valid MCH human resources analysis can also be used as a tool for policy making when managing and delivering MCH health services [12].

In China, MCH was categorized as a specific area where women and children can receive specialized all-round curative and preventive healthcare by health professionals from general hospitals, MCH institutions and community-based healthcare services at province, prefectural, county, township and village level [1, 13], In addition, there are also some auxiliary MCH workers, including specific MCH health education staff and MCH annual report statisticians, the common point of these MCH workers is their working contents, which only focus on MCH [14]. In order to learn the situation of MCH human resources in China, a national survey on MCH human resources was conducted in 2011 and in this study we analyzed the numbers, distribution and capacity of the MCH human resources.

## Methods

### The MCH human resources

In China, health workers should hold health qualification certificates, even for health workers who providing auxiliary health services, such as management and statistics when they work in health institutions [15]. In this study, the MCH human resources were defined as workers who 1) provide MCH-related services and 2) hold at least one legal health qualification certificate. All other workers were excluded.

In this study, MCH-related services include:

1. Women’s health: female adolescent health care, female pre-marital health care, preconception care, basic obstetric care, emergency obstetric care, maternal health care, postpartum health care, menopause and elderly health care, women’s mental health care, women’s nutrition and general gynecological diseases screening.
2. Children’s health: basic pediatric health care, neonatal emergency care, neonatal disease screening, newborn hearing screening, breastfeeding guidance, growth and nutritional care, mental health care, oral health care, hearing health care, eye health care, children’s intellectual development inspection, children physical examination, children rehabilitation.
3. MCH health education: preparing MCH health educational materials, community and institution-based health education, matrimony health education and pregnancy education.
4. MCH training and supervision: supervision and training of junior staff and medical students.
5. MCH management: monitoring the quality of MCH work; management and monitoring of MCH health related archives.
6. MCH related research projects: international and domestic special projects on MCH.
7. MCH statistics: monitoring, collecting and analyzing MCH information for annual reports; reporting the annual report quality control results; maternal, child mortality assessment.

### Sampling of the MCH-related institutions

The Chinese Government has established a hierarchical administrative network for MCH, which can provide a full range of healthcare services covering the entire health care services spectrum for women and children [1]. The top-down sequence is province level, prefectural level, county level, township level and village level [13]. In this study, we chose cities in prefectural level as the sampling unit level since the sampling size at a higher level (province level) would not have been enough, whereas sampling at prefectural level can cover all levels and types of medical and health care institutions providing MCH services. This study is a national institution-based sampling survey with formal approval from both the national maternal and child health annual report office and the National Health and Family Planning Commission of the People’s Republic of China (the former Chinese Ministry of Health) [16, 17] . All data was anonymized and we were granted permission to use and analyze the data by all the participating institutions.

**Fig. 1:**
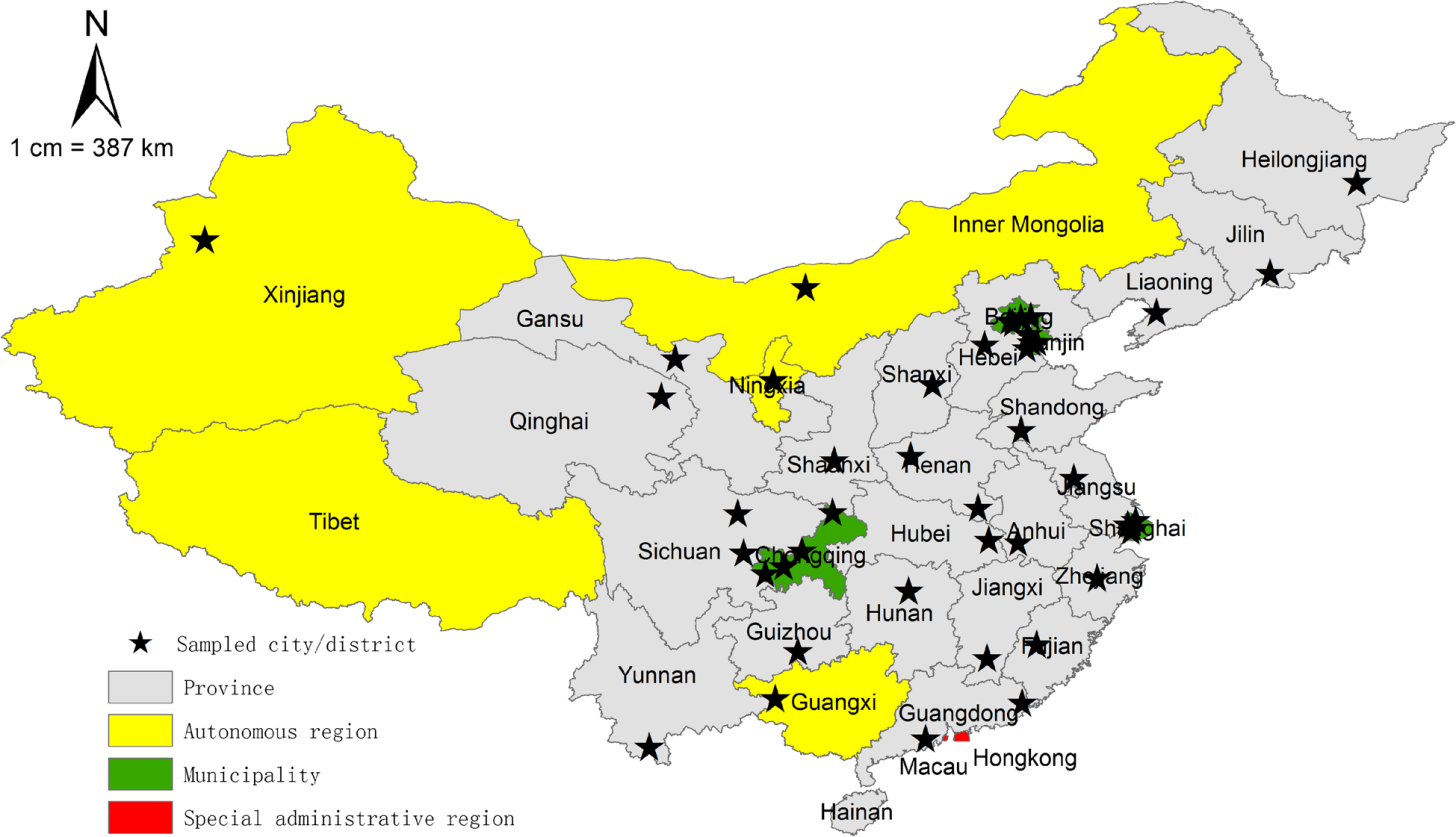
Map of China showing the 22 provinces, 5 autonomous regions, 4 municipalities and selected samples

For the 22 provinces and 5 autonomous regions in China, 28 prefectural-level districts/cities were selected out of all the 332 municipality prefectural districts/cities by random cluster sampling. Then, for the four municipalities (Beijing, Shanghai, Tianjin and Chongqing) in China, two urban districts and two rural counties were drawn respectively by random cluster sampling. Finally, all the selected districts/cities covered all province level administrative divisions in China except for Tibet autonomous region, Hainan province (there are only a few cities) and the two special administrative regions (Hong Kong and Macau), the distribution of the sampled districts/cities is shown in Fig. 1. Then all randomly selected districts/cities were treated as clusters and all the medical and health care institutions providing MCH services were selected as our targeted institutions.

### Procedures

 A structured questionnaire, which was developed and improved by several rounds of expert consultation and then piloted in a study, was used in this institution-based survey. Questions on MCH human resources were mainly about the demographic characteristics, the amount and types of provided services and the education level and skills of the health professionals; Questions on MCH-related institutions were mainly about the type and location of the institution. The questionnaires were sent to the institutions by post with assistance of local health administrative departments. Then they were completed by the institutions’ heads under the supervision of their local technicians, who had already received training in Beijing (especially in relation to classification of the different service types). Questionnaires were then sent back to the research group directly. Finally, we received all the data from every health institution, and the participating institution heads granted us permission to use the data, all the data was anonymized and none of the researchers could identify any personal information.

### Statistical analysis

Data were weighted based on the proportion of sampled districts/cities. The weights were 4 for Beijing and Tianjin, 4.5 for Shanghai, 10 for Chongqing and 11 for all other cities (see Appendix 1 for more details). By using the weights, the numbers of the weighted populations (permanent resident population) in the sampled districts/cities were very near (5.7 % error) to the actual census populations in 2010. Then data were grouped into three geographic regions: east, central and west, which are defined by the Chinese Government and roughly represent different levels of economic development. East region areas are the most developed, whereas the west region is the least developed. The east region includes 11 provinces: Beijing, Tianjin, Hebei, Liaoning, Shanghai, Jiangsu, Zhejiang, Fujian, Shandong, Guangdong and Hainan; the central region includes 8 provinces: Shanxi, Jilin, Heilongjiang, Anhui, Jiangxi, Henan, Hubei and Hunan; and the west region includes 12 provinces: Inner Mongolia, Guangxi, Shaanxi, Gansu, Qinghai, Ningxia, Xinjiang, Sichuan, Chongqing, Guizhou, Yunnan and Tibet [18].

When counting the number of MCH workers, a weight 0.5 was assigned to workers who were not working full-time in MCH to avoid overestimation of the total MCH human resources. The total number per 10,000 population and proportion of MCH human resources were calculated in order to reveal the overall quantity of the MCH human resources in China. Stratified calculations by different regions, different levels and types of health institutions were also presented. When evaluating the capacity of the MCH services, three proxies were chosen: % of appropriately educated of health workers, % of health workers that could perform cardiopulmonary resuscitation (as a proxy of common medical skill), and for women’s health workforce, % of medical staff that could perform caesarean section, forceps and vacuum extraction deliveries (as a proxy of maternal health related skill). Therefore, we only focused on the workers providing curative and preventive women and children’s healthcare services in the MCH frontline. Other auxiliary MCH service (health education, training and supervision, management, project/ research/ education, statistics) providers were excluded because these services can also be effectively provided by less educated health workers.

All analyses were conducted in the software SPSS 13.0.

## Results

### Quantity of Chinese MCH human resources

There were 77,248 MCH workers in the investigated 5,168 health institutions in 2010. The number of MCH workers per 10,000 population was 5.5 at national level, 5.7 for the east and west regions, and 5.1 for the central region. Most MCH workers were female (Table 1), especially for workers providing women’s healthcare services (95.2 %), health education (90.2 %) and statistics (90.4 %). The gender distributions were similar across all regions.

**Table 1:** Percentage of female MCH workers for the different service types in the three regions

	Type of MCH work (%)						
Region	Women’s health	Children’s health	Health education	Training and supervision	Management	Project/ research/ education	Statistics
East	95.2	77.8	89.7	87.7	78.6	86.0	89.2
Central	94.3	72.9	89.3	86.4	74.0	82.9	89.4
West	96.4	83.7	91.6	90.4	83.6	89.0	92.4
Total	95.2	78.2	90.2	88.2	78.7	86.2	90.4

*Abbreviation*: *MCH* maternal and child health

40.5 % of the MCH workforce concentrated on women’s healthcare services followed by children’s curative and preventive healthcare services (25.7 %) and MCH education (15.5 %; Table 2). The proportions of MCH workforce for other service types (training, supervision, management, research and statistical information management) were near 10 %.

**Table 2:** Service distribution of MCH workers in the three regions

		Type of MCH work (n(%))						
Region	Number of MCH human resources (n)	Women’s health	Children’s health	Health education	Training and supervision	Management	Project/ research/ education	Statistics
East	296225	110535(37.3)	63895(21.6)	38882(13.1)	30156(10.2)	26210( 8.8)	21453(7.2)	25448( 8.6)
Central	256426	112805(44.0)	66759(26.0)	41360(16.1)	30327(11.8)	26477(10.3)	15818(6.2)	26675(10.4)
West	226614	92042(40.6)	69742(30.8)	40510(17.9)	31123(13.7)	26553(11.7)	18754(8.3)	26218(11.6)
Total	779265	315382(40.5)	200396(25.7)	120752(15.5)	91606(11.8)	79240(10.2)	56025(7.2)	78341(10.1)

*Abbreviation*: *MCH* maternal and child health

At national level, 48.8 % of the MCH workers were working in general hospitals (accounting for 21.4 % of all health institutions) and 26.7 % in specialized MCH institutions (5.6 % of all health institutions). 59.4 % of all health institutions were township or community health centers but only 23.0 % MCH workers worked there (Table 3). Higher percentages of MCH workers worked in general hospitals and community health centers in the east region, while the percentage of MCH workers in township health centers was much higher in the central region.

**Table 3:** Institutional distribution of MCH workers in the three regions

		Type of MCH institutions (n(%))					
Region	Number of MCH human resources (n)	General hospital	Specialized MCH institution	Township health center	Community health center	Family planning service station	Others
East	296225	162509(54.9)	74841(25.3)	41374(14.0)	15820(5.3)	727(0.2)	956(0.3)
Central	256426	103947(40.5)	69611(27.1)	64345(25.1)	10724(4.2)	7205(2.8)	594(0.2)
West	226614	113604(50.1)	63292(27.9)	36551(16.1)	10167(4.5)	2530(1.1)	470(0.2)
Total	779265	380059(48.8)	207744(26.7)	142270(18.3)	36710(4.7)	10462(1.3)	2020(0.3)

*Abbreviation*: *MCH* maternal and child health

Most of the Chinese MCH workers (44.2 %; Table 4) were working in county level health institutions (accounting for 20.64 % of all MCH institutions), and the proportions of MCH workers in prefectural level (5.22 % of all MCH institutions) and township level (73.19 % of all MCH institutions) MCH-related institutions were 23.4 % and 24.7 % respectively. Only 0.94 % of the MCH-related institutions were at provincial level and 7.7 % of the total MCH workers were placed in these institutions. In general, 1/16 of all the MCH-related institutions were located in provincial and prefectural cities but accounted for 1/3 of all the MCH workers. Each provincial level MCH-related institution employed nearly 117 MCH workers. The corresponding numbers were 64 for prefectural level and 31 for county level health institution, and only less than 5 for township level.

**Table 4:** Locational distribution of MCH workers in the three regions

		Location level of MCH institutions (n(%))			
Region	Number of MCH human resources (n)	Province level	Prefectural level	County level	Township level
East	291563	19751(6.8)	82978(28.5)	126614(43.4)	62221(21.3)
Central	256349	3762(1.5)	45994(17.9)	126363(49.3)	80230(31.3)
West	226603	35772(15.8)	52442(23.1)	89301(39.4)	49088(21.7)
Total	774515	59285(7.7)	181414(23.4)	342277(44.2)	191540(24.7)

*Abbreviation*: *MCH* maternal and child health

### Capacity of Chinese MCH human resources

#### Education levels

The education levels of women’s and children’s curative and preventive healthcare personnel are shown in Tables 5 and 6. In general, 67.6 % and 71.9 % of the women’s and children’s health care professionals had an associate degree or higher. Only 1.6 % and 2.3 % of the women’s and children’s health care professionals had masters or higher degrees respectively. The proportion of MCH workers with high education level (including bachelor, master and above) was largest in the east provinces, and lowest in the central region. In general hospitals and specialized MCH institutions, MCH workers with master and higher degrees were relatively more than in other health institutions. In township health centers and community health centers, those with high level education accounted for around 40.0 %. The proportion of MCH workers with higher degrees was higher in higher level institutions, for example in the provincial level of MCH-related institutions of the central region, more than 1/3 of the MCH workers hold master or higher education degrees, and those with bachelor and above degrees were more than 80 %.

**Table 5:** Education levels of women and children’s health workers

		Education level(women’s health) (%)				Education level(children’s health) (%)			
Area	Type of MCH-related institutions	Master and above	Bachelor	Associate degree	High school and lower	Master and above	Bachelor	Associate degree	High school and below
East	General hospital	3.7	35.0	33.7	27.7	6.6	41.1	29.8	22.5
	Specialized MCH institution	3.5	38.4	27.8	30.3	2.3	35.4	31.7	30.6
	Township health center/Community health center	0.2	17.6	42.2	40.0	0.3	20.1	41.6	38.0
	Family planning service station	0.0	26.4	31.0	42.6	0.0	0.0	50.0	50.0
	Others	3.0	45.1	23.9	28.0	0.0	65.5	19.6	14.9
	subtotal	2.3	29.1	35.7	32.9	3.4	31.8	34.8	30.0
Central	General hospital	2.0	29.4	43.1	25.5	1.5	36.3	40.3	21.9
	Specialized MCH institution	0.1	23.7	41.2	35.0	0.2	28.5	45.5	25.8
	Township health center/Community health center	0.0	7.2	45.1	47.7	0.0	8.6	48.1	43.3
	Family planning service station	0.0	12.0	54.2	33.9	0.0	0.0	60.0	40.0
	Others	0.0	12.5	42.5	45.0	0.0	10.3	44.8	44.8
	subtotal	0.6	17.5	44.0	37.8	0.5	20.3	45.4	33.8
West	General hospital	3.7	32.9	45.7	17.8	4.6	35.8	44.7	14.9
	Specialized MCH institution	0.2	20.0	48.9	30.9	0.2	27.8	46.7	25.3
	Township health center/Community health center	0.0	13.5	50.0	36.6	0.0	13.8	49.5	36.8
	Family planning service station	0.0	22.3	41.7	35.9	0.0	37.5	62.5	0.0
	Others	0.0	6.3	68.8	25.0	0.0	22.2	44.4	33.3
	Subtotal	2.0	24.9	47.4	25.7	2.8	28.6	46.3	22.3
Total	General hospital	3.2	32.7	40.9	23.1	4.6	37.4	39.5	18.4
	Specialized MCH institution	1.4	28.2	38.1	32.4	0.9	30.4	41.6	27.1
	Township health center/Community health center	0.1	12.3	45.4	42.3	0.1	13.6	46.5	39.8
	Family planning service station	0.0	16.2	48.5	35.3	0.0	17.6	58.8	23.5
	Others	0.9	21.0	42.2	35.9	0.0	26.2	39.1	34.7
Total		1.6	23.7	42.3	32.3	2.3	27.0	42.6	28.2

*Abbreviation*: *MCH* maternal and child health

**Table 6:** Education levels of women and children’s health workers of different institutions in the three regions

		Education level(women’s health) (%)				Education level(children’s health) (%)			
Area	Location level of MCH-related institutions	Master and above	Bachelor	Associate degree	High school and lower	Master and above	Bachelor	Associate degree	High school and below
East	Province level	16.4	44.5	17.1	21.9	14.8	38.5	27.1	19.6
	Prefectural level	4.5	41.9	28.3	25.3	10.9	42.1	29.9	17.2
	County level	2.4	35.2	32.9	29.5	1.3	39.3	31.7	27.8
	Township level	0.2	17.7	42.3	39.8	0.3	21.0	40.9	37.7
	Subtotal	2.2	29.0	35.7	33.1	3.4	31.7	34.9	30.0
Central	Province level	33.9	46.4	14.3	5.4	40.0	40.0	16.0	4.0
	Prefectural level	2.5	41.3	36.6	19.5	1.5	45.1	35.2	18.3
	County level	0.2	21.9	45.5	32.4	0.1	27.4	46.9	25.6
	Township level	0.0	7.6	44.9	47.5	0.0	9.1	47.6	43.3
	Subtotal	0.6	17.6	44.0	37.8	0.5	20.3	45.4	33.8
West	Province level	7.2	37.3	43.4	12.1	6.2	39.5	44.8	9.5
	Prefectural level	3.3	38.2	43.0	15.5	5.1	34.9	44.3	15.8
	County level	0.4	22.3	50.0	27.2	0.2	30.0	47.4	22.5
	Township level	0.2	13.3	49.4	37.1	0.1	13.8	48.6	37.5
	Subtotal	2.0	24.9	47.4	25.7	2.8	28.6	46.3	22.3
Total	Province level	9.9	39.0	37.5	13.6	9.5	39.2	38.7	12.6
	Prefectural level	3.3	40.1	37.5	19.1	5.0	38.3	40.1	16.6
	County level	1.1	27.1	41.8	29.9	0.6	32.6	41.3	25.5
	Township level	0.1	12.5	45.1	42.3	0.1	14.1	45.8	39.9
Total		1.6	23.7	42.3	32.4	2.2	27.0	42.6	28.2

*Abbreviation*: *MCH* maternal and child health

#### Professional skills

The proportions of women’s health workers capable of caesarean section, forceps and vacuum extraction deliveries were estimated across regions, types and levels of health institutions (Table 7). In general, more than 40 % of the women’s health workers were capable of providing these delivery services, and those with vacuum extraction delivery skills were more than 50 %. The proportion of women’s health workers who could provide skilled birth attendance (including caesarean section, forceps and vacuum extraction deliveries) was largest in the central region, while the east and the west had similar proportions. In terms of different types of health institutions by regions, the proportions of MCH workforces who could provide skilled birth attendance were highest in general hospitals for all regions. Family planning service stations were the second and specialized MCH institutions were the third in the east and the central regions. However, in the west region, proportions of skilled birth attendants were relatively low (less than 10 %) in the family planning stations, whereas the proportions of skilled birth attendants were similar across general hospitals and specialized MCH institutions. In terms of distribution by location levels of health institutions, the proportions of skilled birth attendants were similar among prefectural and county level health institutions, ranking the first, and then followed by provincial level and township level MCH-related institutions.

**Table 7:** Proportions of the MCH workers who can deliver or conduct cardiopulmonary resuscitation of different institutions in the three regions

		Proportion of women’s workers who can do (%)			Proportion of conducting cardiopulmonary resuscitation (%)	
Area	Type of MCH-related institutions	Cesarean	Forceps Delivery	Vacuum extraction delivery	Women’s health workers	Children’s health workers
East	General hospital	48.8	47.1	57.4	91.2	94.0
	Specialized MCH institution	43.7	43.0	48.2	68.2	66.8
	Township health center/Community health center	32.3	37.4	51.9	80.0	75.9
	Family planning service station	45.5	47.4	64.9	97.5	100.0
	Others	10.0	15.5	32.0	71.9	89.9
	Subtotal	41.8	42.6	53.5	82.5	82.9
Central	General hospital	55.9	56.4	61.5	86.7	90.3
	Specialized MCH institution	46.3	46.9	50.3	86.4	89.3
	Township health center/Community health center	40.5	41.4	55.0	83.1	79.0
	Family planning service station	51.6	53.8	54.4	74.0	100.0
	Others	42.5	42.5	42.5	62.5	55.2
	Subtotal	46.8	47.5	55.9	84.5	84.1
West	General hospital	44.5	47.6	52.3	94.8	96.0
	Specialized MCH institution	45.0	47.4	53.7	82.7	74.3
	Township health center/Community health center	25.0	32.0	41.4	82.0	74.5
	Family planning service station	6.4	3.8	5.1	23.1	100.0
	Others	12.5	12.5	25.0	37.5	33.3
	Subtotal	37.7	42.0	48.3	88.0	87.5
Total	General hospital	48.8	49.6	56.2	91.5	94.4
	Specialized MCH institution	45.0	45.6	50.3	78.9	78.4
	Township health center/Community health center	33.7	37.5	50.3	81.8	76.7
	Family planning service station	38.8	39.9	41.9	63.1	100.0
	Others	28.1	29.4	36.2	59.8	54.9
Total		42.2	44.1	52.6	85.0	85.1

*Abbreviation*: *MCH* maternal and child health

More than 80 % of women and children’s health workers had the capacity to perform cardiopulmonary resuscitation (Table 8). In terms of different types of health institutions, the proportions of health workers capable of cardiopulmonary resuscitations were higher than 90 % in general hospitals, but were also high in specialized MCH institutions, township health center and community health care centers (more than 75 %). Provincial level institutions employed the highest proportions of skilled cardiopulmonary resuscitation health workers (above 95 %), followed by prefectural levels (higher than 90 %). County level and township level MCH-related institutions had similar proportions which were all above 75 %.

**Table 8:** Proportions of MCH workers who can deliver or conduct cardiopulmonary resuscitation of different location levels

		Proportion of women’s workers who can do (%)			Proportion of conducting cardiopulmonary resuscitation (%)	
Area	Level of medical and health care units	Cesarean	Forceps Delivery	Vacuum extraction delivery	Women’s health workers	Children’s health workers
East	Province level	46.7	35.7	43.9	96.6	99.4
	Prefectural level	46.4	41.0	54.3	81.3	83.9
	County level	46.7	46.9	54.7	84.0	85.1
	Township level	33.8	38.5	53.0	80.2	75.8
	Subtotal	41.6	42.5	53.6	82.6	82.9
Central	Province level	61.5	69.2	69.2	95.0	100.0
	Prefectural level	42.6	45.4	48.2	92.8	93.1
	County level	55.0	54.8	59.3	83.7	88.4
	Township level	40.8	41.6	54.9	82.8	78.8
	Subtotal	46.8	47.5	55.8	84.5	84.0
West	Province level	40.2	49.6	51.9	99.2	97.8
	Prefectural level	50.3	49.6	55.6	94.8	97.2
	County level	41.2	43.4	49.7	85.3	81.0
	Township level	25.3	31.8	40.7	81.3	74.6
	Subtotal	37.7	42.0	48.3	88.0	87.5
Total	Province level	41.6	47.4	50.7	98.6	98.4
	Prefectural level	47.1	46.3	53.0	90.8	94.4
	County level	48.2	48.8	54.9	84.2	85.0
	Township level	34.5	38.0	50.5	81.6	76.6
Total		42.1	44.1	52.6	85.0	85.1

*Abbreviation*: *MCH* maternal and child health

## Discussion

Our study is the first nationwide MCH human resources investigation in China. In this study, the situation of MCH human resources in 2010 was evaluated by analyzing the quantity, capacity and distribution of MCH workers. The health institution samples covered a large range of areas and had a relatively symmetrical distribution. The four municipalities were sampled separately, so the corresponding samples could represent the four municipal cities. But for the other areas, the sampling representativeness for each province cannot be guaranteed; therefore we cannot use the samples to estimate the MCH human resources in each province or autonomous district. We can however make a good assessment for the whole nation and the three regions (East, West and Central China). The samples were drawn at prefectural level and all types and location levels of health institutions providing MCH services in the sampled areas were investigated. To account for the sampling error when drawing samples from the prefectural level, we weighted the data according to the sampling proportions of each region.

This survey found that 5.5 MCH workers per 10,000 population were available in 2010. The National Health and Family Planning Commission of the People’s Republic of China recommended that the MCH human resources to population allocation standard should be 1:10,000 in general, 1: 5,000 in less populated areas and 1: 15,000 in populated areas respectively [14]. According to this threshold, our findings suggest that the Chinese MCH human resources in 2010 were sufficient. However, China is a large and unevenly developing country, a rough conclusion of “enough MCH human resources” is apparently inappropriate, the situation may be different or even inverse in poor rural areas. However, detailed MCH human resource information at provincial level could not be estimated in this study because of our study design limiting our ability to examine inequities across these levels. Further studies exploring the difference of MCH human resources among rural, urban, populated, and less populated areas should be conducted and new MCH human resource thresholds for regions with different population density and developing levels should also be set by the policy makers to suit the different situation in different areas.

This study revealed the uneven distribution of both the quantity and capacity of the MCH human resources. This situation is incompatible with the national primary health care policy, which recommends that basic clinical and preventive care services and the corresponding human resources should evenly distribute in all levels of health institutions and not concentrated only in higher level general hospitals. Therefore measures should be introduced to allocate more MCH professionals in lower level institutions and in less-developed areas. Motivating and encouraging policies such as reasonable remuneration and corresponding reward systems should be developed. In addition, only recruiting new professionals cannot solve the problem but sometimes may result in a waste of human resources, several measurements, such as further training, effective working arrangement, etc. can be taken to improve current workers’ skills.

There are some limitations in this study. The International Standard Classification of Occupations (ISCO) was not adopted in this study. MCH human resources were classified based on the types of MCH service activities, so there might be some difficulties to conduct external comparison with other similar research results in other countries, and internal comparison among different health professions, such as between nurses and midwives. The three indicators for representing MCH capacity provided an opportunity to have a glance of the overall capacity of MCH human resources, and different distribution among different developing-level regions and institutions. However, it’s also difficult to compare between different professional groups (such as, doctors vs. nurses) because all professionals were mixed in the analysis, which also make it hard to judge the overall capacity of MCH human resources because of its limited external comparability and the lack of national MCH service capacity regulation or estimation thresholds.

According to the development plan for women and children (2011–2020), the Chinese government put an emphasis on expanding the MCH human resources [19], however, based on the results of our study, adding new MCH workers is not a solution to the MCH human resource situation, more investigations on MCH human resources should be taken to provide useful and detailed evidence-based information for policy makers to accelerate the evenly MCH development in China.

## Conclusions

The MCH human resources in China are not evenly distributed among the different institutions and regions, and around 30 % of MCH workers have only a high-school degree or lower. Similar is the situation for the whole health workforce in China [20]. Therefore, more workers with higher education degrees need to be reallocated to improve the MCH services in China.

## Appendix 1

Weighting Method

Because the sampling unit is districts/cities at prefectural level (including prefectural cities in province and districts in municipalities), so the weights are mainly based on the proportion of sampled districts/cities:

For Beijing, there are 16 districts, and we selected 4, so the weight was 16/4 = 4

For Tianjin, there are 16 districts, and we selected 4, so the weight was 16/4 = 4

For Shanghai, there are 15 districts, and we selected 4, so the weight was 18/4 = 4.5

For Chongqing, there are 15 districts, and we selected 4, so the weight was 40/4 = 10

For all provinces and autonomous regions, there are total 332 prefectural level cities, from which we select 28, so the weight should be 332/28 = 11.8, but we decreased it to 11 by experts consulting and research experience.

Finally, by using the weights, the results turned out to be good, the sum of the weighted populations in the sample districts/cities was very near (with only 5.7 % errors) to the actual population number (China census 2010).

## Abbreviations

MCH: maternal and child health
MDG: Millennium Development Goal
